# Family Conflict and Neighborhood Context in Latino Adolescent Depression: An ABCD Study

**DOI:** 10.21203/rs.3.rs-10527707/v1

**Published:** 2026-08-05

**Authors:** Micaela Lembo, Giselle A. Barreto, Angela R. Laird, Mariana Sanchez, Zoran Bursac, Diana D. Sheehan, Gladys E. Ibanez

**Affiliations:** Florida International University Robert Stempel College of Public Health and Social Work; Florida International University Robert Stempel College of Public Health and Social Work; Florida International University; Florida International University Robert Stempel College of Public Health and Social Work; Florida International University Robert Stempel College of Public Health and Social Work; Florida International University Robert Stempel College of Public Health and Social Work; Florida International University Robert Stempel College of Public Health and Social Work

## Abstract

Latino adolescents experience disproportionately high rates of depressive symptoms relative to other racial and ethnic groups in the United States, yet the multilevel factors shaping these disparities remain understudied. Family conflict is an important predictor of adolescent mental health, particularly within Latino communities where family relationships occupy a central role in social and emotional development. Using data from the Adolescent Brain Cognitive Development (ABCD) Study four-year follow-up (N = 1,796), we examined whether neighborhood social vulnerability moderated the association between family conflict and depressive symptoms among Latino adolescents. Multilevel models were estimated separately for each of the four Social Vulnerability Index (SVI) themes: socioeconomic status, household composition/disability, minority status/language, and housing/transportation. Family conflict was a consistent predictor of depressive symptoms across all four models (Theme 1: b = 5.36, p < .001; Theme 2: b = 4.91, p < .001; Theme 3: b = 3.00, p = .159; Theme 4: b = 3.60, p = .015), with a mean CBCL withdrawn-depressed T-score of 53.96 (SD = 6.49) across the sample. No interaction effects were statistically significant. These findings underscore the centrality of family dynamics as a driver of depressive symptoms among Latino adolescents regardless of neighborhood context. Future research should integrate opportunity-focused neighborhood metrics, investigate mediation pathways, and incorporate additional ecological factors to better understand the multilevel determinants of Latino adolescent mental health.

## Introduction

Latino adolescents have experienced elevated and increasing rates of depressive symptoms compared to other racial and ethnic groups in the U.S., with the largest increase in major depressive episodes of any group between 2009 and 2019.^1^ More than 30% of Latino adolescents currently report depressive symptoms, yet most existing research has focused on clinical samples, recently immigrated youth, or geographically limited populations, limiting generalizability to the broader U.S. Latino adolescent population.^–5^ Family conflict is a well-established predictor of adolescent depressive symptoms and emotional distress, and its consequences for Latino adolescents extend beyond emotional suffering to academic underachievement, substance use, and elevated suicide risk.^1,6–8^ Because family relationships occupy a central role in Latino cultural values, family conflict may carry particular weight for adolescent mental health in this population.^9,10^

Neighborhood conditions also shape adolescent mental health, with socioeconomic disadvantage, housing instability, and limited community resources linked to depressive symptoms and psychological distress.^11,12^ Latino families are more likely to reside in disadvantaged neighborhoods due to longstanding residential segregation.^13–15^ Therefore neighborhood-level factors may compound family-level stressors affecting adolescent mental health, making neighborhood context a particularly salient determinant of health in this population.^13–15^ However, few studies have examined how neighborhood vulnerability interacts with family environments to influence depressive symptoms among Latino adolescents using large, population-based datasets.

Guided by the Mental Health and Well-being Socio-Ecological Model, which theorizes that health outcomes are shaped by interacting influences across multiple ecological levels, the present study examined whether neighborhood social vulnerability moderates the association between family conflict and depressive symptoms among Latino adolescents using data from the Adolescent Brain Cognitive Development (ABCD) Study.^16^ Within this framework, family dynamics operate at the interpersonal level while neighborhood conditions represent a more distal community-level influence, suggesting that neighborhood context may amplify or attenuate the impact of family conflict on adolescent mental health. We hypothesized that greater neighborhood social vulnerability would strengthen the association between family conflict and depressive symptoms, such that adolescents in more vulnerable neighborhoods would experience greater depressive symptoms in the context of family conflict.

## Methods

### Participants

This study used a cross-sectional, with the primary exposure and outcome measured concurrently at the ABCD Study four-year follow-up. data from the Adolescent Brain Cognitive Development (ABCD) Study were used for this analysis, the largest longitudinal study of brain development and child health in the United States.^17^ Enrollment began in 2016 and included approximately 11,800 youth aged 9–10 years recruited across 21 sites nationwide, with eligibility requiring residence within a 50-mile radius of an ABCD Study site.^18^ All study procedures were approved by the IRB at each participating site and by a centralized IRB at the University of California, San Diego; caregivers provided written informed consent and children provided informed assent. Detailed recruitment and assessment procedures have been described previously.^17,18^ This study was pre-registered on the Open Science Framework (OSF; https://osf.io/4mrva/).

ABCD Study data are publicly available through the NIH Brain Development Cohorts Data Hub (https://www.nbdc-datahub.org/). This study utilized ABCD Release 6.1 (https://doi.org/10.82525/ab4q-qr87), specifically the four-year follow-up visit, selected because key analytic variables were collected exclusively at this time point, when adolescents were approximately 13–15 years of age.

### Measures

#### Caregiver-Reported Demographics

Caregiver-reported demographic data from baseline (youth/caregiver race, ethnicity, nativity status, primary language) and four-year follow-up (adolescent age and gender, caregiver education) were used in this analysis. Demographic characteristics of the analytic sample are presented in Table 1.

#### Adolescent-Reported Conflicted Family Environment Scale

##### Adolescent-Reported Conflicted Family Environment Scale

The Family Environment Scale (FES) is a widely used measure designed to assess the social and emotional characteristics of family environments.^19^ The full scale consists of 90 items across multiple subscales evaluating key dimensions of family functioning, including cohesion, expressiveness, conflict, organization, and control. For the present study, only the conflict subscale was used. This nine-item subscale assesses family tension and discord by measuring the frequency of arguments, unresolved disputes, and emotional strain among family members. Items are scored dichotomously (true/false) and averaged across the nine items to yield a mean conflict score ranging from 0 to 1, with higher scores indicating a more conflict-ridden family environment. In previous studies, the subscale has demonstrated acceptable internal consistency, with Cronbach’s alpha ranging from 0.63 to 0.68. Reliability of the FES conflict subscale has also been supported in Latino samples, with caregiver-rated alpha coefficients ranging from .68 to .86 across studies of Latino families, including a Spanish-language adaptation.^20^ 41 Adolescent-reported FES conflict scores collected at the four-year follow-up served as the primary exposure variable in this analysis.

#### Caregiver-Reported Child’s Behavior Checklist

The Child Behavior Checklist (CBCL) is a widely used caregiver-report questionnaire designed to assess emotional, behavioral, and social functioning in children and adolescents.^21^ The full scale consists of 113 items evaluating a broad range of behavioral and emotional problems over the preceding six months, which are combined to generate internalizing (e.g., anxiety, depression), externalizing (e.g., aggression, rule-breaking), and total problem scores. For the present study, only the withdrawn/depressed syndrome subscale was used, which measures symptoms related to sadness, social withdrawal, and loss of interest in activities. Scores are standardized as T-scores, with higher scores indicating greater depressive symptomatology. In previous studies, this subscale demonstrated good internal consistency (Cronbach’s alpha = 0.82).^21^ Caregiver-reported CBCL withdrawn/depressed T-scores collected at the four-year follow-up served as the primary outcome in this analysis.

#### Neighborhood Factors

Neighborhood social vulnerability was assessed using the 2016 Social Vulnerability Index (SVI), a metric developed by the Centers for Disease Control and Prevention (CDC) using U.S. Census data.^22,23^ The SVI measures the relative community vulnerability to external stressors across four themes: (1) socioeconomic status, (2) household characteristics, (3) racial and ethnic minority status, and (4) housing type and transportation. Each theme comprises multiple census-tract level variables capturing distinct aspects of community vulnerability. SVI scores are calculated as percentile rankings from 0 to 1, with higher values indicating greater vulnerability relative to other census tracts within the same state and were treated as continuous variables in all models.

### Analyses

#### Multilevel Modeling

The analytic sample was restricted to participants identified as Hispanic or Latino at baseline, based on caregiver report. Participants were excluded for missing/unknown ethnicity data, missingness exceeding 50% across analytic variables, or missing data on either primary variable (FES conflict subscale or CBCL withdrawn/depressed subscale). Of the approximately 11,800 ABCD participants, 2,411 were identified as Hispanic/Latino at baseline and 1,913 completed the four-year follow-up (498 lost to follow-up). From this Year 4 sample, 82 were excluded for missing exposure/outcome data and 35 for missing site or family identifier data, yielding a final analytic sample of 1,796 participants.

Multilevel modeling (MLM) was used to examine the association between adolescents’ perceived family conflict and depressive symptoms within the context of neighborhood social vulnerability. This approach was selected to account for the hierarchical structure of the ABCD dataset, in which participants were nested within families and sites. A random intercept for family and site was assessed as possible hierarchical clustering variables. The primary exposure was adolescent-reported FES conflict subscale scores collected at the four-year follow-up. The primary outcome was caregiver-reported CBCL withdrawn/depressed T-scores also collected at the four-year follow-up. Neighborhood context was operationalized using the four SVI theme scores measured at baseline.

Models were built progressively across four steps. First, an unconditional model was estimated to partition the variance in depressive symptoms across families, from which the intraclass correlation coefficient (ICC) was calculated to quantify the proportion of variance attributable to family-level differences. Second, individual-level predictors were added, including FES conflict scores and relevant covariates. Third, neighborhood-level SVI theme scores were introduced. Finally, cross-level interaction terms between FES conflict scores and each SVI theme score were included to test whether neighborhood social vulnerability moderated the association between family conflict and depressive symptoms. Separate models were estimated for each of the four SVI themes. Model assumptions including linearity, independence of errors, and homoscedasticity were evaluated. Model fit was compared using − 2 Log Likelihood, Akaike Information Criterion (AIC), and Bayesian Information Criterion (BIC).

Adjusted models included the following covariates: child sex, child age, primary language spoken at home, parent race, parent education, and child nativity status. Covariates were included as fixed effects and were selected based on theoretical relevance to adolescent depressive symptoms and family functioning.

All multilevel models were estimated in R (version 4.6.0; R Core Team, 2026) using the lme4 package (version 2.0–1; Bates et al., 2015). Multiple imputation was implemented using the mice package (version 3.19.0; van Buuren & Groothuis-Oudshoorn, 2011).^24–26^

#### Missing Data

The extent and patterns of missingness were first examined across all analytic variables using descriptive statistics and visualizations to assess the proportion and distribution of missing values. Multiple imputation was used to address missing data, consistent with best practices for large-scale longitudinal studies.^18^ Imputation was implemented using the mice package in R, which applies multiple imputation by chained equations (MICE) to generate plausible values for missing observations based on the observed data.^19^ Ten imputed datasets were generated using a maximum of 20 iterations per imputation cycle. Imputed datasets were analyzed separately and pooled using Rubin’s Rule to reduce potential bias and improve statistical efficiency.

## Results

### Sample Characteristics

The analytic sample consisted of 1,796 Hispanic/Latino adolescents from the ABCD Study four-year follow-up and can be found in Tables 1 and 2. The sample was 53% male with a mean age of 14.0 years (SD = 0.7). Most participants reported English as their primary language (64%), with 35% reporting Spanish. Regarding race, 57% identified as White, 23% as other race, and 15% as multiracial. Parent education was distributed across categories, with the largest proportion reporting some college or associate degree (34%), followed by bachelor’s degree (19%) and less than high school (18%). Most adolescents were US-born (94%). Recruitment sites were distributed across 17 states, with the largest proportions from California (39%) and Florida (20%).

### Descriptive Statistics

Descriptive statistics for continuous key variables are presented in Table 3. The mean family conflict score was 0.25 (SD = 0.23), indicating generally low levels of perceived family conflict across the sample. The mean CBCL withdrawn-depressed T-score was 53.96 (SD = 6.49), slightly above the normative mean of 50. SVI theme scores varied across themes, with Theme 3 (minority status/language) showing the highest mean percentile (M = 0.76, SD = 0.24), reflecting the high concentration of minority and non-English-speaking residents in neighborhoods where this Latino sample resided. SVI data were missing for 162 participants across all four themes.

### Multilevel Modeling

All models were estimated across 10 multiply imputed datasets and polled using Rubin’s rules. Prior to estimating the primary models, an unconditional model was fit to partition variance in CBCL withdrawn-depressed T-scores across levels. The site-level ICC was approximately 1%, indicating minimal clustering at the recruitment site level and was removed from further modeling. The family-level ICC was 28.7%, indicating that approximately 28.7% of the variance in depressive symptoms was attributable to differences between families, supporting the presence of meaningful family-level clustering and justifying the multilevel modeling approach.

Results from the four adjusted multilevel moderation models are presented in Table 4. Family conflict was a significant positive predictor of depressive symptoms in three of the four models. Specifically, higher family conflict was associated with significantly higher CBCL withdrawn-depressed T-scores in the socioeconomic theme model (B = 5.36, SE = 1.44, p < .001), the household composition/disability theme model (B = 4.91, SE = 1.38, p < .001), and the housing/transportation theme model (B = 3.60, SE = 1.48, p = .015). The association was in the same positive direction but did not reach statistical significance in the minority status/language theme model (B = 3.00, SE = 2.13, p = .159).

The main effect of neighborhood social vulnerability was not significant in any of the four models (all p > .05), suggesting that neighborhood vulnerability alone was not directly associated with depressive symptoms in this sample.

Critically, none of the FES × SVI interaction terms were statistically significant across the four theme models (Theme 1: B = − 4.51, SE = 2.32, p = .053; Theme 2: B = − 4.35, SE = 2.68, p = .105; Theme 3: B = − 0.13, SE = 2.78, p = .964; Theme 4: B = − 1.18, SE = 2.46, p = .632), indicating that neighborhood social vulnerability did not significantly moderate the association between family conflict and depressive symptoms.

## Discussion

This study examined whether neighborhood social vulnerability moderated the association between family conflict and depressive symptoms among Latino adolescents using data from the ABCD Study four-year follow-up. Family conflict was a robust and consistent predictor of depressive symptoms across all four SVI theme models, while neighborhood social vulnerability did not significantly moderate this relationship. No interaction terms reached statistical significance, though the interaction between family conflict and socioeconomic vulnerability (Theme 1) approached significant thresholds (B = − 4.51, p = .053), and the main effect of family conflict was non-significant in the minority status and language model (Theme 3; B = 3.00, p = .159) (Table 4). Importantly, the effect of family conflict on depressive symptoms remained consistent regardless of the broader community context in which adolescents resided, underscoring the centrality of the family environment as one of the primary drivers of depressive symptoms in this population.^6,7,9,10,27–29^ These findings align with the Mental Health and Well-being Socio-Ecological Model, which situates family dynamics at the interpersonal level and neighborhood context at the more distal community level. Because interpersonal-level factors are closer to the individual, they may exert stronger direct effects on mental health than community-level factors, which may help explain why family conflict emerged as the dominant predictor of depressive symptoms independent of neighborhood social vulnerability.^16^ These null findings should not be interpreted as evidence that neighborhood context is irrelevant to adolescent mental health; rather, they may reflect limitations in how neighborhood vulnerability is operationalized.

The SVI captures markers of social marginalization and structural risk but does not necessarily capture the resources and opportunities most relevant to moderating the family conflict-depression relationship among Latino adolescents. Its focus on vulnerability rather than opportunity may limit its utility for detecting neighborhood-level moderation in this context. The null interaction findings also raise the possibility that neighborhood context operates through family dynamics rather than alongside them. Neighborhood stressors such as economic hardship, housing instability, and community violence may contribute to elevated family conflict, which in turn drives depressive symptoms, suggesting a mediation pathway rather than a moderation pathway.^29,13,14^ However, the non-significant main effect of family conflict in the minority status and language model (Theme 3) may reflect the complexity of majority-minority neighborhood contexts, which can be simultaneously protective, fostering social cohesion and cultural connectedness, and risk-amplifying, exposing residents to discrimination, immigration-related stress, and concentrated disadvantage.^14,29–31^ This interplay may account for the attenuated, non-significant association observed in Theme 3.

### Strengths and Limitations:

Various strengths of the present study merit consideration. First, the use of ABCD Study data provided a large, geographically diverse, population-based sample of Latino adolescents drawn from 17 states, offering greater generalizability than clinical or regionally limited samples common in prior research. Second, this study was pre-registered on the Open Science Framework prior to analysis, enhancing transparency and reducing the risk of outcome reporting bias. Third, the multi-informant measurement approach, with adolescents reporting on family conflict and caregivers reporting on depressive symptoms, reduces the potential for same-source bias that can inflate associations when a single reporter provides both exposure and outcome data. Fourth, missing data were addressed using multiple imputation with 10 imputed datasets pooled via Rubin’s rules, consistent with best practices for large-scale longitudinal research. Finally, neighborhood social vulnerability was examined across all four SVI themes in separate models, providing a more nuanced assessment of neighborhood context than a single composite score would allow.

Several limitations should be considered when interpreting these findings. First, neighborhood social vulnerability was measured at a single baseline timepoint using residential addresses available only at baseline. This cross-sectional measurement approach does not capture cumulative or sustained exposure to neighborhood disadvantage across development, which may have led to an underestimation of neighborhood effects on adolescent mental health outcomes. Second, because residential addresses were only available at baseline, adolescents who relocated during the follow-up period would still be assigned their baseline neighborhood characteristics; given that adolescents were approximately 13–14 years old at the four-year follow-up, residential mobility during this period cannot be ruled out, and the resulting misclassification of residential context may have contributed to the null findings observed in the interaction models. Third, household income and parent nativity status could not be included as covariates due to missingness, limiting the ability to fully account for socioeconomic and generational context. Finally, ABCD Study site was used as a proxy for geographic clustering in multilevel models. As participants were recruited based on residence within a 50-mile radius of a study site, site-level clustering may not fully capture the neighborhood-level variation in social vulnerability experienced by participants at follow-up.

### Future Directions

Future research should prioritize examining neighborhood context through a more expansive lens by incorporating measures such as the Child Opportunity Index (COI), which captures the resources and opportunities available to children rather than vulnerability alone.^32–34^ This shift in measurement would better align with the Healthy People 2030 social determinants of health framework and provide a more comprehensive understanding of how neighborhood conditions shape mental health outcomes among Latino adolescents.^35,36^ Additionally, future studies should consider testing mediation pathways to examine whether neighborhood context operates through family conflict to influence depressive symptoms, rather than alongside it. Incorporating other ecological levels, including school environment, peer relationships, and cultural values such as familismo, within a socio-ecological framework would provide a more complete picture of the multilevel factors shaping depressive symptoms in this population. Finally, longitudinal designs that capture residential mobility and changes in neighborhood context over time would address key measurement limitations identified in the present study and strengthen causal inference in this area of research.

## Conclusion

Family conflict remains a consistent predictor of depressive symptoms among Latino adolescents across all neighborhood vulnerability contexts; underscoring the centrality of family dynamics in shaping mental health outcomes in this population. These findings highlight the need for family-focused clinical and public health interventions targeting Latino youth. While neighborhood social vulnerability did not moderate this relationship, future work should continue to identify the neighborhood characteristics that may promote resilience rather than reflect only disadvantage.

## Figures and Tables

**Table 1:** Sample characteristics of Latino Year 4 participants (ABCD) in the multilevel analytic sample (N = 1,796).

Characteristic	Overall n = 1796
**Child sex, n (%)**	
Male	952 (53.0%)
Female	844 (47.0%)
**Child age (years), Mean (SD)**	14.0 (0.7)
**Primary household language, n (%)**	
English	1,145 (64.0%)
Spanish	628 (35.0%)
Other	23 (1.3%)
**Race, n (%)**	
White	1,026 (57%)
Black	63 (3.5%)
American Indian / Alaska Native	18 (1.0%)
Other race	417 (23.0%)
Multiracial	264 (15.0%)
Unknown	8 (0.4%)
**Parent education, n (%)**	
Less than high school	314 (18.0%)
High school/GED	269 (15.0%)
Some college/Associate	609 (34.0%)
Bachelor’s	346 (19.0%)
Graduate/Professional	253 (14.0%)
Unknown	5 (0.3%)
**Child US-born, n (%)**	
US-born	1,686 (94.0%)
Non–US-born	105 (5.9%)
Unknown	5 (0.3%)

*Note*. Hispanic/Latino subsample with Year 4 assessments and complete data on CBCL withdrawn-depressed syndrome score, family conflict (Family Environment Scale), recruitment site (state), and family ID (N = 1,796). CBCL = Child Behavior Checklist. Categorical variables: n (% of non-missing); continuous: mean (SD). US-born defined using ABCD country code for the United States.

**Table 2:** Recruitment sites (ABCD study site host state) in the multilevel analytic sample (N = 1,796).

Site (state)	n (%)
**Recruitment site (state), n (%)**	
California	697 (39%)
Colorado	97 (5.4%)
Connecticut	111 (6.2%)
Florida	366 (20%)
Maryland	30 (1.7%)
Michigan	49 (2.7%)
Minnesota	28 (1.6%)
Missouri	22 (1.2%)
New York	34 (1.9%)
Oklahoma	124 (6.9%)
Oregon	77 (4.3%)
Pennsylvania	9 (0.5%)
South Carolina	12 (0.7%)
Utah	68 (3.8%)
Vermont	9 (0.5%)
Virginia	35 (1.9%)
Wisconsin	28 (1.6%)

*Note*. Same complete-case analytic sample as Table 1 (N = 1,796). Values are n (% of sample) by recruitment site host state.

**Table 3:** Descriptive statistics for continuous key variables (multilevel analytic sample; N = 1,796).

Variable	Mean (SD) [min, max]
**Family conflict (FES mean)**	0.25 (0.23) [0.00, 1.00]
**CBCL withdrawn–depressed T-score**	53.96 (6.49) [50.00, 93.00]
**SVI theme 1 percentile**	0.58 (0.29) [0.00, 1.00]
Unknown	162
**SVI theme 2 percentile**	0.44 (0.26) [0.01, 1.00]
Unknown	162
**SVI theme 3 percentile**	0.76 (0.24) [0.02, 1.00]
Unknown	162
**SVI theme 4 percentile**	0.56 (0.28) [0.00, 1.00]
Unknown	162

*Note*. Same analytic sample as Table 1 (N = 1,796). FES = Family Environment Scale (higher = more conflict). CBCL = Child Behavior Checklist; T-scores are normed (M = 50, SD = 10 in the ABCD reference sample). SVI = CDC/ATSDR Social Vulnerability Index; theme percentiles 0–100 at census-tract level (primary address). Theme 1 = socioeconomic; 2 = household composition/disability; 3 = minority status/language; 4 = housing/transportation. Bracketed values are observed min and max (non-missing). Descriptives are not pooled across multiply-imputed data sets; regression results in Table 4 use multiple imputation.

**Table 4:** Multilevel moderation models: association between family conflict and depressive symptoms by SVI theme (N = 1,796).

Family conflict (FES mean)	Theme 1: Socioeconomic	Theme 2: Household composition / disability	Theme 3: Minority status / language	Theme 4: Housing / transportation
	**5.36 (1.44), p < .001**	**4.91 (1.38), p < .001**	**3.00 (2.13), p = .159**	**3.60 (1.48), p = .015**
**Main effect of SVI theme percentile**	0.13 (0.93), p = .891	1.21 (0.94), p = .198	−1.52 (1.16), p = .188	0.88 (0.89), p = .324
**Family conflict × SVI theme**	−4.51 (2.32), p = .053	−4.35 (2.68), p = .105	−0.13 (2.78), p = .964	−1.18 (2.46), p = .632
**Random effects**				
**Family variance**	9.432	9.275	8.961	8.941
**Residual variance**	29.745	29.971	30.270	30.324

*Note*. Outcome is the CBCL withdrawn-depressed syndrome T-score (higher = more symptoms). Each column is a separate adjusted model: outcome regressed on family conflict (FES mean), the SVI theme, and their interaction, covariates, and a random intercept for family, fit in each imputed data set. Fixed effects for family conflict, the SVI theme, and their interaction are pooled with Rubin’s rules (m = 10). Covariate slopes are omitted from the table. Random-intercept and residual variances and AIC/BIC are arithmetic means across the m fitted models (descriptive). Models adjusted for child sex; Highest parent/caregiver education level; child country of birth; child age at visit; child native language; recruitment site host state; parent/caregiver race (collapsed). FES = Family Environment Scale. Mean AIC across imputations: Theme 1 = 11862.9, Theme 2 = 11866.5, Theme 3 = 11866.6, Theme 4 = 11868.2. Mean BIC across imputations: Theme 1 = 12384.8, Theme 2 = 12388.4, Theme 3 = 12388.5, Theme 4 = 12390.1.
